# Assessment of prognostic value of preoperative neutrophil-to-lymphocyte ratio for postoperative mortality and morbidity

**DOI:** 10.3389/fmed.2023.1102733

**Published:** 2023-03-08

**Authors:** Yingchao Zhu, Yaodan Bi, Bin Liu, Tao Zhu

**Affiliations:** ^1^Department of Anesthesiology, West China Hospital, Sichuan University, Chengdu, Sichuan, China; ^2^Department of Anesthesiology, Peking Union Medical College, Peking Union Medical College Hospital, Chinese Academy of Medical Sciences, Beijing, China

**Keywords:** risk assessment, serum biomarkers, neutrophil-to-lymphocyte ratio, surgery, postoperative outcomes, mortality, subgroup

## Abstract

**Background:**

The preoperative elevated neutrophil-to-lymphocyte ratio (NLR) was reported to be associated with poorer outcomes after cancer and cardiovascular surgeries. It is unclear, however, if the predictive value is particular or if it may be applied to other types of surgery. We aimed to assess the prognostic value of preoperative NLR levels for morbidity and mortality after various surgery and determine an optimal threshold for NLR.

**Methods:**

We conducted a cohort analysis on patients receiving surgery at Sichuan University West China Hospital between 2018 and 2020. Multivariable piecewise regression analysis were used to determine the optimal cutoff value of NLR. Subgroup analysis were performed to verify the correlation. Sensitivity analysis was used to explore the effect of different thresholds.

**Results:**

We obtained data from 136,347 patients. The optimal cutoff of NLR was determined as 3.6 [95% CI (3.0, 4.1)] by piecewise regression method. After multivariable adjustment, preoperative high NLR remained significantly associated with increased in-hospital mortality (aOR, 2.19; 95% CI, 1.90–2.52; *p* < 0.001) and ICU admission after surgery (aOR, 1.69; 95% CI, 1.59–1.79; *p* < 0.001). Subgroup analyses confirmed the predictive value of high NLR in multiple surgical subgroups, including general, orthopedic, neurosurgical, and thoracic surgery subgroups, otorhinolaryngology, head and neck surgery, and burn plastic surgery. A NLR threshold of 3.6 gave excellent predictive value, whether employed alone or added in an extended model.

**Conclusions:**

In conclusion, the association of elevated NLR with higher mortality and ICU admission can be extended to a wider range of procedures. NLR threshold of 3.6 could provide good prognostic value for the prognostic model.

## Introduction

The neutrophil-to-lymphocyte ratio (NLR) is a prognostic marker that reflects systemic inflammation and resulting immunosuppression (1–3). The advantage is that NLR is easily acquired from routine complete blood counts, compared to C-reactive protein and Interleukin-6. This easy and affordable test has become widely used in recent years. Zahorec (4) suggested that NLR correlates with organ dysfunction scores and clinical course in critical patients. In addition, several studies have shown that NLR has an advantage over CRP in predicting prognosis and determining the severity of diseases such as multiple sclerosis and emergency abdominal surgery. Several studies in 2021 (5–7) revealed the role of NLR in the early diagnosis and stratification of COVID-19 patients. Because of its accessibility of use, preoperative biomarker analysis has become a technique for predicting unfavorable postoperative outcomes. Like surgical intervention and postoperative pain, preoperative stress such as preoperative conditions, comorbidities, and psychological stress can also cause changes in NLR.

In cancer procedures, NLR has been proven to be an independent predictor of death and tumor recurrence (1–3, 8, 9). According to a 2015 comprehensive review (10), higher NLR was related with greater long-term mortality and morbidity following major cardiac and vascular operations. Recently, the predictive importance of postoperative NLR in a range of operations, including abdominal surgery (11, 12), orthopedic surgery (13), and bariatric surgery (14), has been revealed. However, due to the small sample population and different definitions of elevated NLR, it is difficult to reach a unified conclusion on the correlation between NLR and surgical prognosis.

In addition to the inflammatory response and immunological alterations produced by surgical intervention, NLR level (15) might reflect patients' prior physical status and comorbidities. Furthermore, the NLR has been linked to not just mortality but myocardial infarction and coronary artery disease (16–18). However, few studies have examined whether the relationship between preoperative NLR and postoperative outcomes can be extended to other types of surgery. Moreover, NLR often uses different cut-off points in various studies.

We aimd to determine whether the relationship between increased NLR and higher mortality could be extended to a wide range of surgeries. And explore the optimal threshold of NLR.

## Methods

### Study design and data collection

The Strengthening the Reporting of Observational Studies in Epidemiology (19) declaration is followed by this retrospective cohort study. Death certificates and medical information were used to determine in-hospital mortality. The ICU electronic information system was used to get ICU admission. The MINS was obtained by postoperative laboratory tests. Due to the sensitive nature of the data used in this study, hospital information center staff members without knowledge collected the data. Independent researchers who were blind to the outcomes compiled the baseline features into a standardized form after obtaining the raw data from the preoperative evaluation sheets. Qualified researchers with experience in human subject confidentiality agreements carried out the data analysis. All data were anonymized and de-identified for confidentiality reasons. The Sichuan University Ethics Committee granted our ethical approval (Project No. 1082 in 2021). The requirement of consent to participate was not required.

We screened all patients over 14 years old who underwent surgery in West China Hospital of Sichuan University from February 2018 to November 2020. The following patients were excluded: (1) Obstetrics, interventional, ophthalmology, and painless diagnosis and treatment procedures and operations; (2) Hospital stay < 24 h (day surgery); (3) Patients without available preoperative NLR measurement before surgery.

The sample size was determined using the Clinical Prediction Model Sample size guidelines (20). With an estimated mortality of 1%, the highest R-squared would be 0.17. The prediction model was estimated to explain 15% of the variability, hence a R-squared of 0.026 was predicted. The shrinkage was set at 2.5%. A minimum of 73,996 samples were required, equal to 814 events and 16 variables.

### Procedures

The primary outcome was in-hospital mortality, with ICU hospitalization and perioperative cardiac injury as secondary events (PMI). In-hospital mortality was defined as death from any cause while in the hospital. ICU admission was defined as being in the ICU for more than 24 h, omitting patients who were in the ICU prior to surgery. PMI was defined as a 14 ng/L or greater absolute rise in peak postoperative hs-cTnT concentrations over baseline. To identify myocardial injury, physicians evaluate high-risk populations based on clinical criteria and expertise. Patients who did not have a postoperative myocardial enzyme assay were presumed to be free of acute myocardial damage.

We also generated a list of risk-adjustment variables, including patient age, sex, body mass index (BMI), 15 preoperative comorbidities, 12 preoperative laboratory tests, 4 prognostic models [ASA, CCI, RCRI, Ex-care (21)], types of surgery, and detail of anesthesia and intraoperative management. Elevated preoperative serum creatinine was defined as > 100 mmol L^−1^ in men and 90 mmol L^−1^ in women. Intraoperative blood transfusion was defined as the infusion of any blood product during surgery. Hemoglobin levels below 120 g L^−1^ for women and 130 g L^−1^ for males were considered preoperative anemia.

### CBC measurements and management

Blood samples collected in EDTA-based anticoagulated tubes yielded fresh blood aliquots. For total blood cell counts and differential leukocyte counts, all blood samples were processed on a Sysmex XN-9000 (TOA Medical Electronics, Kobe, Japan). The blood sample can be tested at room temperature for up to 10 h before degrading and becoming untrustworthy. The sample can also be kept in the refrigerator at −4°C for up to 7 days. NLR was computed by dividing the absolute value of neutrophils by the number of lymphocytes.

### Statistical analysis

We reported demographics, comorbidities, and perioperative management for the entire patient cohort. The Mann-Whitney test or the *t*-test were used to compare differences in continuous data, which were provided as mean SD or median with interquartile range. The χ^2^ or Fisher exact test was used to compare categorical data that were given as numbers (percentages).

Preoperative NLR concentration was first analyzed as a continuous variable. We constructed a multivariable logistic regression model based on preoperative NLR concentrations and added all available covariates. Skewness distribution variables are added into the model after logarithmic transformation. Then, lasso regression was used to filter variables and adjust the complexity of the logistic regression model to reduce overfitting. Only variables with VIF ≤ 10 were input into the model. The final screened variables were used in all subsequent multivariate analyses. We assessed multivariable logistic regression model appropriateness by receiver operating curve (ROC) and calibration curve. A spline fitting curve of the multivariable model was constructed to simulate the potential relationship between outcome and NLR, and a non-linear *P*-value > 0.05 was considered to have a linear relationship. We further applied piecewise linear regression model (22) to calculate the optimal threshold of NLR, and analyzed the threshold effect of NLR.

We then reported patient characteristics with different NLR levels. The elevated NLR group was defined as patients with preoperative NLR levels greater than the optimal cut-off value. Univariate odds ratio (OR) and multivariate-adjusted odds ratio (aOR) were reported for postoperative in-hospital mortality among patients with different NLR levels. Subgroup analyses were conducted in subgroups: sex, age, the American Society of Anesthesiologists Physical Status (ASA-PS), comorbidities, and surgery subspecialties. For every risk-factor subgroup, the respective variables defining the risk factor were removed from the analyses. “Extended model” was calculated by adding the preoperative NLR variable to the score of the three commonly-used clinical models including ASA, Charlson Comorbidity Index (CCI), and Surgical Outcome Risk Tool (SORT). We explored the performance differences between extended models with NLR variables with different thresholds. The discrimination of the prediction models was assessed by the area under the receiver operating characteristic curve (AUROC) (23). The reclassification power was assessed by the net reclassification improvement (NRI) between the extended model corresponding to each threshold and the model corresponding to threshold 3.6 of NLR. The model fit was assessed using the Hosmere-Lemeshow goodness-of-fit test and Akaike Information Criterion (AIC). The Brier score indicates the models' predictive accuracy.

Statistical analyses were performed with R 4.0.2 (Vienna, Austria; http://www.R-project.org/).

## Results

### Baseline characteristics

Supplementary Figure 1 depicts the flow of participants. This study's cohort included 136,347 patients [69,152 men (50.7%)] with a median age of 52 years (range, 40–63 years). The full cohort is detailed in Table 1. The total cohort's median NLR was 2.09 [interquartile range (IQR), 1.54–3.04]. Preoperative NLR was substantially greater in dead patients (median, 6.64; IQR, 2.88–14.01) compared to survivors (median, 2.08; IQR, 1.53–3.00).

**Table 1:** Baseline characteristics of patients.

**Variable**		**level**	**Overall**	**Alive**	**Death**	***P*-value**
*n*	*n*		136,347	134,869	1,478	
Sex [*n* (%)]		Men	69,152 (50.7)	68,222 (50.6)	930 (62.9)	< 0.001
		Women	67,177 (49.3)	66,629 (49.4)	548 (37.1)	
Age [median (IQR)]			52.00 (40.00, 63.00)	52.00 (40.00, 63.00)	58.00 (47.00, 69.00)	< 0.001
BMI [median (IQR)]			23.00 (21.00, 25.00)	23.00 (21.00, 25.00)	23.00 (20.00, 25.00)	< 0.001
Prognostic models	ASA-PS [*n* (%)]	I–II	96,675 (71.0)	96,459 (71.6)	216 (14.6)	< 0.001
		III	37,465 (27.5)	36,727 (27.3)	738 (50.0)	
		IV–V	2,082 (1.5)	1,561 (1.2)	521 (35.3)	
	CCI [*n* (%)]	≤ 4	117,135 (85.9)	115,891 (85.9)	1,244 (84.2)	0.057
		5–8	3,622 (2.7)	3,570 (2.6)	52 (3.5)	
		≥9	15,590 (11.4)	15,408 (11.4)	182 (12.3)	
	RCRI [*n* (%)]	0	773 (0.6)	726 (0.6)	47 (3.2)	< 0.001
		1	70,958 (53.4)	70,282 (53.4)	676 (46.7)	
		2	54,653 (41.1)	54,101 (41.1)	552 (38.1)	
		≥3	6,559 (4.9)	6,386 (4.9)	173 (11.9)	
	Excare [median (IQR)]		13.22 (13.22, 19.88)	13.22 (13.22, 19.88)	24.13 (19.88, 30.79)	< 0.001
Comorbidities [*n* (%)]	Ischemic heart disease	No	132,291 (97.0)	130,921 (97.1)	1,370 (92.7)	< 0.001
		Yes	4,056 (3.0)	3,948 (2.9)	108 (7.3)	
	Atrial fibrillation	No	133,962 (98.3)	132,623 (98.3)	1,339 (90.6)	< 0.001
		Yes	2,385 (1.7)	2,246 (1.7)	139 (9.4)	
	Chronic heart failure or cardiomyopathy	No	136,164 (99.9)	134,714 (99.9)	1,450 (98.1)	< 0.001
		Yes	183 (0.1)	155 (0.1)	28 (1.9)	
	Valvular disease	No	130,622 (95.8)	129,254 (95.8)	1,368 (92.6)	< 0.001
		Yes	5,725 (4.2)	5,615 (4.2)	110 (7.4)	
	Peripheral vascular disease or abdominal aorticaneurysm	No	132,999 (97.5)	131,574 (97.6)	1,425 (96.4)	0.006
		Yes	3,348 (2.5)	3,295 (2.4)	53 (3.6)	
	Hypertension	No	112,095 (82.2)	111,091 (82.4)	1,004 (67.9)	< 0.001
		Yes	24,252 (17.8)	23,778 (17.6)	474 (32.1)	
	Cerebrovascular disease	No	135,503 (99.4)	134,051 (99.4)	1,452 (98.2)	< 0.001
		Yes	844 (0.6)	818 (0.6)	26 (1.8)	
	Hemiplegia paraplegia or paralytic syndrome	No	135,661 (99.5)	134,219 (99.5)	1,442 (97.6)	< 0.001
		Yes	686 (0.5)	650 (0.5)	36 (2.4)	
	Chronic obstructive pulmonary disease	No	130,343 (95.6)	129,035 (95.7)	1,308 (88.5)	< 0.001
		Yes	6,004 (4.4)	5,834 (4.3)	170 (11.5)	
	Diabetes	No	126,767 (93.0)	125,542 (93.1)	1,225 (82.9)	< 0.001
		Yes	9,580 (7.0)	9,327 (6.9)	253 (17.1)	
	Cancer (including lymphoma and leukemi)	No	96,307 (70.6)	95,161 (70.6)	1,146 (77.5)	< 0.001
		Yes	40,040 (29.4)	39,708 (29.4)	332 (22.5)	
	Childpugh grade	A	104,071 (76.3)	103,383 (76.7)	688 (46.5)	< 0.001
		B	32,274 (23.7)	31,484 (23.3)	790 (53.5)	
		C	122,588 (92.2)	121,465 (92.4)	1,123 (77.6)	< 0.001
	Preoperative anemia	No	10,355 (7.8)	10,030 (7.6)	325 (22.4)	
		Yes	129,973 (95.3)	129,028 (95.7)	945 (63.9)	< 0.001
	Preoperative increased creatinine	No	6,374 (4.7)	5,841 (4.3)	533 (36.1)	
		Yes	19,990 (92.5)	19,817 (93.2)	173 (49.3)	< 0.001
	Preoperative leukocytosis (%)	No	1,518 (7.0)	1,372 (6.5)	146 (41.6)	
		Yes	106 (0.5)	74 (0.3)	32 (9.1)	
Emergency case [*n* (%)]	Emergency		126,602 (93.7)	125,940 (94.3)	662 (45.2)	< 0.001
	Elective		8,479 (6.3)	7,675 (5.7)	804 (54.8)	
Surgical category [*n* (%)]	General		44,856 (32.9)	11,101 (8.2)	16 (1.1)	< 0.001
	Orthopedic		21,715 (15.9)	21,633 (16.0)	82 (5.5)	
	Urological		18,141 (13.3)	3,317 (2.5)	26 (1.8)	
	Neurosurgery		11,625 (8.5)	18,116 (13.4)	25 (1.7)	
	Otorhinolaryngology, head and neck		11,117 (8.2)	44,524 (33.0)	332 (22.5)	
	Thoracic		10,504 (7.7)	5,708 (4.2)	129 (8.7)	
	Cardiovascular		9,209 (6.8)	10,964 (8.1)	661 (44.7)	
	Burn and plastic		3,343 (2.5)	9,062 (6.7)	147 (9.9)	
	Other		5,837 (4.3)	10,444 (7.7)	60 (4.1)	
General anesthesia [*n* (%)]		No	7,770 (5.7)	7,715 (5.7)	55 (3.8)	0.002
		Yes	127,872 (94.3)	126,472 (94.3)	1,400 (96.2)	
Intraoperative hypotension [*n* (%)]	MAP < 55 mmHg at any time	No	117,270 (86.0)	116,311 (86.2)	959 (64.9)	< 0.001
		Yes	19,077 (14.0)	18,558 (13.8)	519 (35.1)	
Intraoperative mean heart rate [median (IQR)]			68.31 (62.77, 75.56)	68.25 (62.74, 75.40)	82.10 (69.69, 96.53)	< 0.001
Intraoperative transfusion [median (IQR)]		No	130,797 (95.9)	129,751 (96.2)	1,046 (70.8)	< 0.001
		Yes	5,550 (4.1)	5,118 (3.8)	432 (29.2)	
Duration of surgery [median (IQR)]			90.00 (50.00, 160.00)	90.00 (50.00, 159.00)	180.00 (92.00, 274.00)	< 0.001
Preoperative laboratory tests	Hb [median (IQR)]		135.00 (123.00, 147.00)	135.00 (124.00, 147.00)	123.00 (100.00, 141.00)	< 0.001
	BUN [median (IQR)]		4.90 (4.00, 6.00)	4.90 (4.00, 6.00)	5.60 (4.30, 8.11)	< 0.001
	CRE [median (IQR)]		68.00 (57.00, 81.00)	68.00 (57.00, 80.00)	72.00 (57.00, 94.00)	< 0.001
	eGFR [median (IQR)]		98.64 (86.18, 109.87)	98.68 (86.35, 109.89)	93.00 (68.46, 107.05)	< 0.001
	TBil [median (IQR)]		11.40 (8.60, 15.20)	11.40 (8.60, 15.20)	12.10 (8.40, 18.10)	< 0.001
	ALB [median (IQR)]		44.10 (41.10, 46.80)	44.10 (41.20, 46.90)	39.00 (32.80, 43.10)	< 0.001
	ALT [median (IQR)]		18.00 (13.00, 28.00)	18.00 (13.00, 28.00)	20.00 (14.00, 37.00)	< 0.001
	LDH [median (IQR)]		165.00 (144.00, 191.00)	164.00 (144.00, 191.00)	208.00 (167.00, 280.50)	< 0.001
	ALP [median (IQR)]		75.00 (61.00, 93.00)	75.00 (61.00, 93.00)	80.00 (63.00, 105.00)	< 0.001
	G [median (IQR)]		5.03 (4.64, 5.61)	5.02 (4.64, 5.59)	6.76 (5.19, 9.49)	< 0.001
	NLR [median (IQR)]		2.09 (1.54, 3.04)	2.08 (1.53, 3.00)	6.64 (2.88, 14.01)	< 0.001

BMI, body mass index; ASA-PS, American Society of Anesthesiologists Physical Status; CCI, Charlson Comorbidity Index; RCRI, Revised Cardiac Risk Index; Ex-care, Ex-Care model; MAP, mean arterial pressure; Hb, Hemoglobin; BUN, Blood urea nitrogen; CRE, Creatinine; eGFR, Estimated glomerular filtration rate; TBil, Total bilirubin; ALB, Albumin; ALT, Alanine transaminase; LDH, Lactate dehydrogenase; ALP, Alkaline phosphatase; G, Blood glucose; NLR, neutrophil to lymphocyte ratio.

**Figure 1:**
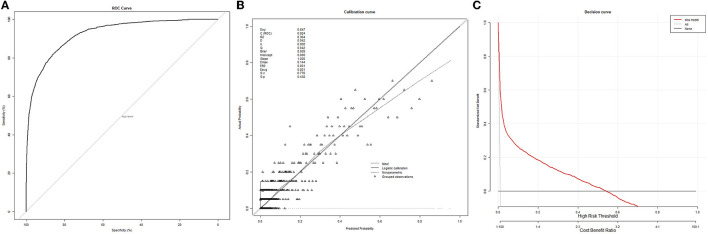
Receiver operating characteristic curves, calibration curve, and decision curve of the multivariate logistic regression model. **(A)** ROC curve. **(B)** Calibration curve. **(C)** Decision curve.

After simplification of lasso regression, we obtained a multivariate logistic regression model consisting of 10 variables (including age, ASA-PS score, emergency surgery, surgical subspecialty, preoperative anemia, preoperative creatinine increase, intraoperative hypotension, intraoperative transfusion, intraoperative mean heart rate, and preoperative NLR). The model's ROC, calibration curve, and decision curve were shown in Figure 1.

**Figure 2:**
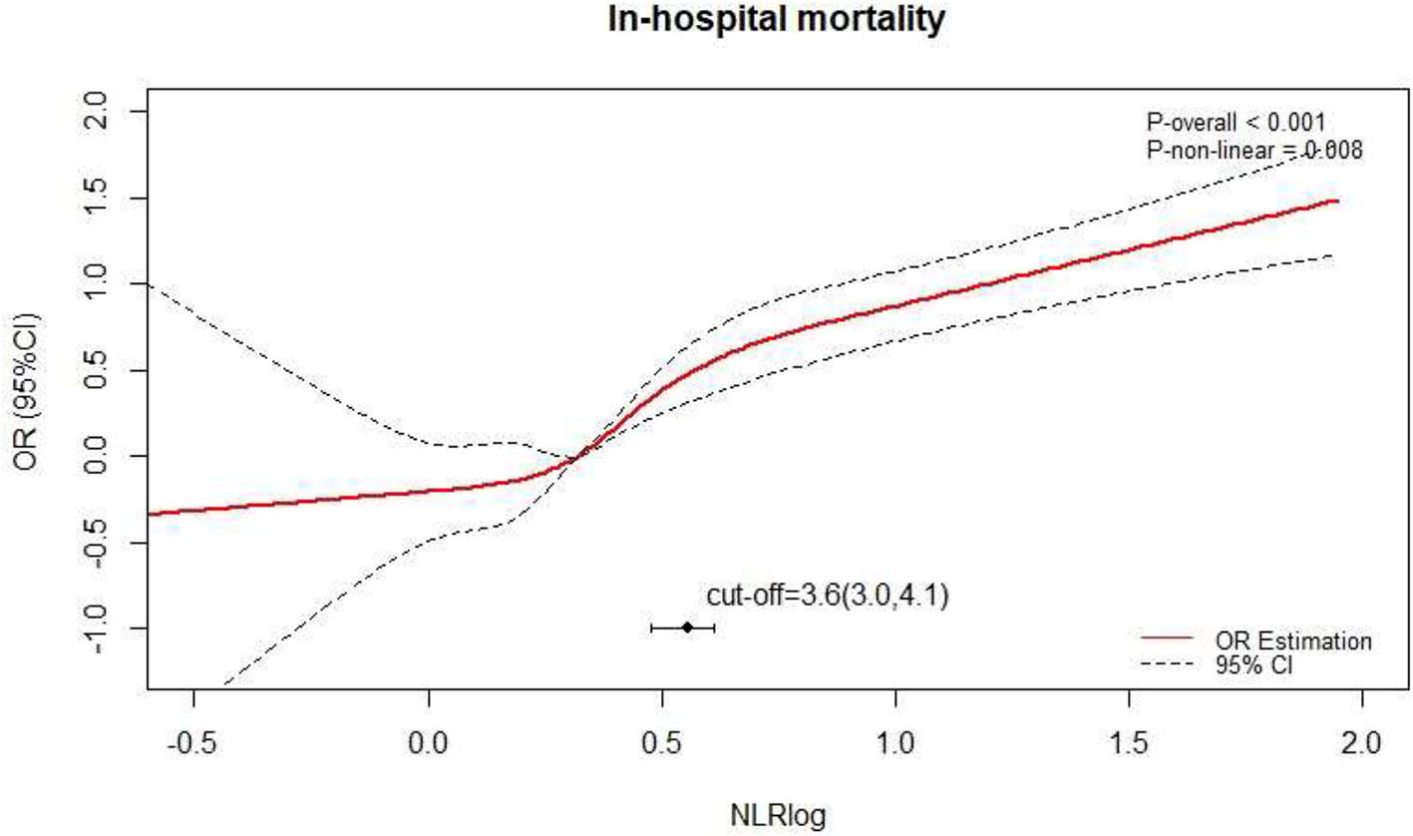
The spline curve of the relationship between preoperative NLR and in-hospital mortality after surgery.

After adjusting for the factors above, a non-linear relationship between NLR and death after surgery was observed (Figure 2; P non-linear < 0.001) by a spline curve. Although the odds ratio gradually increased with the increase of NLR, the overall difference of patients with NLR < 3.6 was small. The optimal truncation point of NLR was determined as 3.6 [95% CI (3.0, 4.1)] by piecewise regression method. The risk of death increased with the logarithmic NLR level up to the turning point (NLR > 3.6) (OR 2.46, 95% CI 2.05–2.94; *p* < 0.001). When the NLR was ≤ 3.6, the logarithmic NLR was not associated with the risk of death (OR 1.42, 95% CI 0.88–2.32; *p* = 0.1) (Table 2).

**Table 2:** Baseline characteristics of patients, according to NLR level.

**Variable**		**Level**	**NLR ≤ 3.6**	**NLR > 3.6**	***P*-value**
*n*	*n*		111,792	245,55	
Sex [*n* (%)]		Men	54,502 (48.8)	14,650 (59.7)	< 0.001
		Women	57,274 (51.2)	9,903 (40.3)	
Age [median (IQR)]			51.00 (40.00, 63.00)	53.00 (42.00, 65.00)	< 0.001
BMI [median (IQR)]			23.00 (21.00, 26.00)	23.00 (20.00, 25.00)	< 0.001
Prognostic models	ASA-PS [*n* (%)]	I–II	84,446 (75.6)	12,229 (49.9)	< 0.001
		III	26,765 (24.0)	10,700 (43.6)	
		IV–V	491 (0.4)	1,591 (6.5)	
	CCI [*n* (%)]	≤ 4	96,520 (86.3)	20,615 (84.0)	< 0.001
		5–8	12,515 (11.2)	3,075 (12.5)	
		≥9	2,757 (2.5)	865 (3.5)	
	RCRI [*n* (%)]	0	59,908 (54.9)	11,050 (46.5)	< 0.001
		1	43,896 (40.2)	10,757 (45.2)	
		2	4,842 (4.4)	1,717 (7.2)	
		≥3	511 (0.5)	262 (1.1)	
	Excare [median (IQR)]		13.22 (13.22, 17.47)	19.88 (13.22, 22.58)	< 0.001
Comorbidities [*n* (%)]	Ischemic heart disease	No	108,712 (97.2)	23,579 (96.0)	< 0.001
		Yes	3,080 (2.8)	976 (4.0)	
	Atrial fibrillation	No	110,022 (98.4)	23,940 (97.5)	< 0.001
		Yes	1,770 (1.6)	615 (2.5)	
	Chronic heart failure or cardiomyopathy	No	111,696 (99.9)	24,468 (99.6)	< 0.001
		Yes	96 (0.1)	87 (0.4)	
	Valvular disease	No	107,108 (95.8)	23,514 (95.8)	0.739
		Yes	4,684 (4.2)	1,041 (4.2)	
	Peripheral vascular disease or abdominal aorticaneurysm	No	109,199 (97.7)	23,800 (96.9)	< 0.001
		Yes	2,593 (2.3)	755 (3.1)	
	Hypertension	No	93,824 (83.9)	18,271 (74.4)	< 0.001
		Yes	17,968 (16.1)	6,284 (25.6)	
	Cerebrovascular disease	No	111,191 (99.5)	24,312 (99.0)	< 0.001
		Yes	601 (0.5)	243 (1.0)	
	Hemiplegia paraplegia or paralytic syndrome	No	111,538 (99.8)	24,123 (98.2)	< 0.001
		Yes	254 (0.2)	432 (1.8)	
	Chronic obstructive pulmonary disease	No	107,378 (96.1)	22,965 (93.5)	< 0.001
		Yes	4,414 (3.9)	1,590 (6.5)	
	Diabetes	No	104,560 (93.5)	22,207 (90.4)	< 0.001
		Yes	7,232 (6.5)	2,348 (9.6)	
	Cancer (including lymphoma and leukemi)	No	78,385 (70.1)	17,922 (73.0)	< 0.001
		Yes	33,407 (29.9)	6,633 (27.0)	
	Childpugh grade	A	90,442 (80.9)	13,629 (55.5)	< 0.001
		B	21,349 (19.1)	10,925 (44.5)	
		C	102,541 (93.9)	20,047 (84.3)	< 0.001
	Preoperative anemia	No	6,616 (6.1)	3,739 (15.7)	
		Yes	111,094 (99.4)	18,879 (76.9)	< 0.001
	Preoperative increased creatinine	No	698 (0.6)	5,676 (23.1)	
		Yes	16,804 (96.0)	3,186 (77.7)	< 0.001
	Preoperative leukocytosis (%)	No	683 (3.9)	835 (20.4)	
		Yes	26 (0.1)	80 (2.0)	
Emergency case [*n* (%)]	Emergency		108,817 (98.3)	17,785 (73.0)	< 0.001
	Elective		1,913 (1.7)	6,566 (27.0)	
Surgical category [*n* (%)]	General		36,720 (32.8)	8,136 (33.1)	
	Orthopedic		16,825 (15.1)	4,890 (19.9)	
	Urological		15,452 (13.8)	2,689 (11.0)	
	Neurosurgery		8,398 (7.5)	3,227 (13.1)	
	Otorhinolaryngology, head and neck		10,057 (9.0)	1,060 (4.3)	
	Thoracic		9,335 (8.4)	1,169 (4.8)	
	Cardiovascular		7,539 (6.7)	1,670 (6.8)	
	Burn and plastic		3,041 (2.7)	302 (1.2)	
	Other		4,425 (4.0)	1,412 (5.8)	
General anesthesia [*n* (%)]		No	6,177 (5.6)	1,593 (6.5)	< 0.001
		Yes	105,101 (94.4)	22,771 (93.5)	
Intraoperative hypotension [*n* (%)]	MAP < 55 mmHg at any time	No	96,948 (86.7)	20,322 (82.8)	< 0.001
		Yes	14,844 (13.3)	4,233 (17.2)	
Intraoperative mean heart rate [median (IQR)]			67.62 (62.37, 74.27)	72.48 (65.32, 82.55)	< 0.001
Intraoperative transfusion [median (IQR)]		No	109,120 (97.6)	21,677 (88.3)	< 0.001
		Yes	2,672 (2.4)	2,878 (11.7)	
Duration of surgery [median (IQR)]			89.00 (49.00, 151.00)	114.00 (60.00, 195.00)	< 0.001
Preoperative laboratory tests	Hb [median (IQR)]		136.00 (126.00, 148.00)	128.00 (110.00, 142.00)	< 0.001
	BUN [median (IQR)]		4.90 (4.00, 5.90)	5.10 (4.00, 6.70)	< 0.001
	CRE [median (IQR)]		67.00 (57.00, 80.00)	69.00 (57.00, 86.00)	< 0.001
	eGFR [median (IQR)]		98.99 (87.25, 109.78)	97.01 (79.90, 110.15)	< 0.001
	TBil [median (IQR)]		11.30 (8.70, 14.90)	12.00 (8.50, 17.20)	< 0.001
	ALB [median (IQR)]		44.40 (41.70, 47.00)	41.90 (37.40, 45.70)	< 0.001
	ALT [median (IQR)]		18.00 (13.00, 28.00)	19.00 (13.00, 31.00)	< 0.001
	LDH [median (IQR)]		162.00 (143.00, 187.00)	179.00 (152.00, 219.00)	< 0.001
	ALP [median (IQR)]		74.00 (61.00, 91.00)	80.00 (64.00, 102.00)	< 0.001
	G [median (IQR)]		4.97 (4.62, 5.44)	5.57 (4.89, 6.89)	< 0.001
	NLR [median (IQR)]		1.88 (1.44, 2.43)	5.40 (4.23, 8.71)	< 0.001
Outcomes	ICU admission (%)	No	102,662 (91.8)	19,904 (81.1)	< 0.001
		Yes	9,130 (8.2)	4,651 (18.9)	
	PMI (%)	No	110,700 (99.0)	24,096 (98.1)	< 0.001
		Yes	1,092 (1.0)	459 (1.9)	
	Length of hospital stay [median (IQR)]		7.00 (4.00, 9.00)	9.00 (5.00, 15.00)	< 0.001
	Death (%)	No	111,337 (99.6)	23,532 (95.8)	< 0.001
		Yes	455 (0.4)	1,023 (4.2)	

NLR, neutrophil to lymphocyte ratio; BMI, body mass index; ASA-PS, American Society of Anesthesiologists Physical Status; CCI, Charlson Comorbidity Index; RCRI, Revised Cardiac Risk Index; Ex-care, Ex-Care model; MAP, mean arterial pressure; Hb, Hemoglobin; BUN, Blood urea nitrogen; CRE, Creatinine; eGFR, Estimated glomerular filtration rate; TBil, Total bilirubin; ALB, Albumin; ALT, Alanine transaminase; LDH, Lactate dehydrogenase; ALP, Alkaline phosphatase; G, Blood glucose; PMI, perioperative myocardial injury.

The patients were divided into groups based on the ideal threshold of 3.6. The low NLR group had 111,792 patients, while the high NLR group included 24,555 patients. The two groups' baseline characteristics and preoperative laboratory testing were comparable (Table 3). However, the prognostic model scores were significantly higher in the High-NLR group. The High-NLR group had a higher proportion of emergency cases (27.0 vs. 1.7%; *P* < 0.001), intraoperative transfusion (11.7 vs. 2.4%; *P* < 0.001), and chronic comorbidities such as chronic heart failure, cardiomyopathy, hemiplegia paraplegia, and paralytic syndrome. There was no significant difference in the proportion of different surgical categories between the two groups, except that the High-NLR group had a higher proportion of neurosurgical procedures (13.1 vs. 7.5%; *P* < 0.001). The duration of surgery was significantly different between the two groups (low NLR: 89.0 vs. high NLR: 114 min; *P* < 0.001). Perioperative mortality (4.2 vs. 0.4%; *P* < 0.001), ICU admission (18.9 vs. 8.2%; *P* < 0.001), and PMI (1.9 vs. 1.0%; *P* < 0.001) were higher in the high NLR group. The length of hospital stay (LOS) was significantly different between the two groups (low NLR: 7.0 vs. high NLR: 9.0 days; *P* < 0.001).

**Table 3:** Threshold effect analysis of NLR on mortality after surgery using piecewise linear regression.

**The optimal threshold of NLR**	**Odds ratio^a^ (95% CI)**	***P*-value**
≤ 3.6	1.42 (0.88, 2.32)	0.100
>3.6	2.46 (2.05, 2.94)	< 0.001

^a^Adjusted: age, ASA-PS score, emergency surgery, surgical subspecialty, preoperative anemia, preoperative creatinine increase, intraoperative hypotension, intraoperative transfusion, intraoperative mean heart rate.

Preoperative high NLR was significantly associated with postoperative in-hospital mortality (OR, 10.6; 95% CI, 9.52–11.9; *p* < 0.001), PMI (OR, 2.14; 95% CI, 1.91–2.39; *p* < 0.001), ICU admission after surgery (OR, 2.63; 95% CI, 2.53–2.73; *p* < 0.001), and LOS (OR, 2.38; 95% CI, 2.32–2.44; *p* < 0.001). After multivariable adjustment, preoperative high NLR remained significantly associated with increased in-hospital mortality (aOR, 2.19; 95% CI, 1.90–2.52; *p* < 0.001) and ICU admission after surgery (aOR, 1.69; 95% CI, 1.59–1.79; *p* < 0.001). Supplementary Figure 2 shows the nomogram of multivariate model with NLR as a categorical variable.

### Sub-group analysis

**Figure 3:**
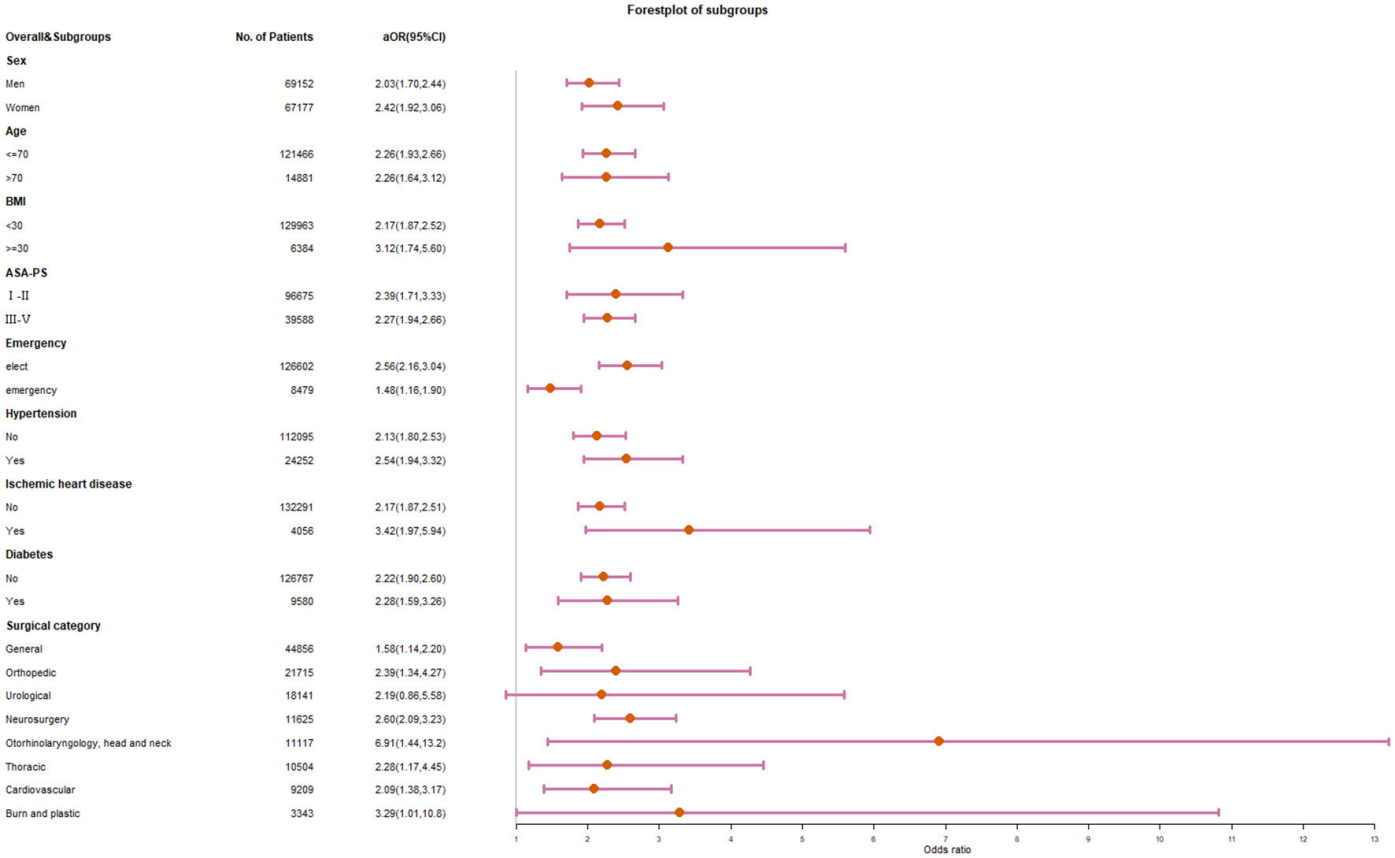
Forest plot for subgroup analysis of preoperative NLR and in-hospital mortality after surgery.

The odds ratio remained stable but showed different effects in different subgroups (Figure 3). There was no significant difference in the correlation between NLR and death among different gender, ages, and ASA subgroups. NLR was more strongly associated with death in the high BMI subgroup (OR 3.12 vs. 2.17) and in elective surgery subgroup (OR 2.56 vs. 1.48). The association was higher in patients with ischemic heart disease, but not significantly higher in patients with hypertension or diabetes. Besides the cardiovascular surgery subgroup, the high NLR group also had a stable OR of 1.58–2.60 in the general, orthopedic, neurosurgical, and thoracic surgery subgroups. The risk of death in patients with high NLR was significantly higher in the Otorhinolaryngology, head and neck surgery (OR 6.90), and burn plastic surgery (OR 3.29). There was an increasing trend of postoperative death in the preoperative NLR group after urological surgery, but it was not statistically significant compared with the normal NLR group.

### Sensitivity analysis

ROC analysis showed that the optimal threshold was 3.91. High preoperative NLR (>3.91) were significantly associated with increased in-hospital mortality (aOR, 2.02; 95% Cl, 1.75–2.32; *p* < 0.001). In the subgroups of cardiovascular surgery and non-cardiovascular surgery, the thresholds of the piecewise regression analysis were 3.33 (95% CI, 1.74–4.92) and 3.53 (95% Cl, 3.02–4.05), respectively. When these thresholds were applied separately, mortality was significantly higher in the high-NLR group than in the low-NLR group between patients with cardiovascular surgery (aOR, 1.75; 95% Cl, 1.16–2.66; *p* < 0.001) and non-cardiovascular surgery (aOR, 2.45; 95% Cl, 2.11–2.85; *p* < 0.001). Supplementary Table 1 shows the performance statistics between the extended models with different NLR threshold. Extended models with NLR variable (threshold 3.6) provided better performance than models with NLR variable (threshold 3 or 10), including greater discrimination (higher AUROC), better reclassification ability (NLR < 0), higher prediction accuracy (lower Brier) and better model fit (lower AIC).

## Discussion

In this study, we found that the preoperative NLR level was associated with postoperative mortality and morbidity in non-cardiac surgery. The optimal preoperative NLR threshold of 3.6 was determined by the piecewise regression method. Subgroup analyses confirmed the predictive value of high NLR in multiple surgical subgroups in addition to cardiovascular surgery.

This study broadens the application of NLR to predict surgical outcomes, demonstrates its role in a broader range of surgical outcomes beyond cardiovascular surgery (24–26), and validates its advantages in conjunction with commonly used clinical models. Various surgical prognostic models can try to incorporate NLR into the screening range of predictors to increase the performance of models. Moreover, the choice of NLR threshold in previous studies is different, which makes it difficult to compare or combine each study. The lack of a unified threshold makes it difficult for clinicians to evaluate NLR quickly, which also hinders the clinical use of NLR. This study included a large number of clinical samples and used the currently recognized multi-factor corrected segmented function to reach the best cut-off point of preoperative NLR, and it still showed significant advantages compared with the two thresholds previously used. Therefore, we suggest that 3.6 might be used as a unified threshold in the future application of preoperative NLR in surgical prognosis research. This study found that the subgroups of general, neurosurgery, orthopedics, and thoracic surgery could also obtain clinically significant correlations. In particular, more studies are needed to explain the mechanism of the high correlation in burns plastic surgery and Otorhinolaryngology, head and neck surgery. Our results suggest that NLR has a stronger effect in patients with chronic diseases that are not easily controlled, such as ischemic heart disease and obesity. Differences in NLR effects on chronic diseases may be due to drug-related long-term remission that changes the association. Previous studies have demonstrated the predictive value of NLR in elective and emergency patients (27, 28), respectively. In this study, subgroups of elective and emergency patients were analyzed, and the results showed that NLR had a higher prognostic correlation in elective patients. In clinical practice, NLR is a dynamic continuous variable rather than a dichotomous variable. Gibson et al. (29) examined the effect of NLR as a continuous variable in the vascular surgery literature, firstly. Although previous studies have shown that increased NLR is associated with adverse outcomes, a consistent cutoff value has not been established (10, 24–26). In order to exclude the influence of confounding factors, we chose to use a piecewise linear regression model to select the best cut-off value instead of roc threshold analysis as in former studies (24, 30).

### Strength and limitation

First, we included a large number of samples and factors to demonstrate the correlation between NLR levels and surgical outcomes, and analyzed the prognostic value of NLR in patients with different types of surgery and different chronic diseases. Secondly, we used the spline function and piecewise regression method instead of univariate ROC to determine a uniform threshold of 3.6 for NLR and surgical prognosis, and the AUROC of the multivariate model could reach 92.4%. This study also has some limitations. First, there is selection bias as a result of the use of retrospective data from a single center. Second, some unadjusted variables, such as the ECG, myocardial enzyme, and the effects of surgery and perioperative treatment, may still result in residual confounding bias. Third, this study found that NLR in patients with obesity, chronic diseases, and burns and plastic surgery may have a higher correlation with prognosis, but the related mechanism needs further study. And our results can only help clinicians to identify potential chronic adverse states or determine the quality of chronic disease control, not to intervene accordingly.

## Conclusion

In conclusion, the association of elevated NLR with higher mortality and ICU admission can be extended to a wider range of procedures. NLR threshold of 3.6 could provide good prognostic value for the prognostic model.

## Data availability statement

The datasets presented in this article are not readily available because this dataset was not publicly available due to ethics committee requirements. Requests to access the datasets should be directed to liubinhxyy@163.com.

## Ethics statement

The Sichuan University Ethics Committee granted our ethical approval (Project No. 1082 in 2021). Written informed consent from the participants' legal guardian/next of kin was not required to participate in this study in accordance with the national legislation and the institutional requirements.

## Author contributions

YCZ: data analysis, interpretation, writing the first draft, revising the first draft, reviewing, and submitting the manuscript. YDB: data collection, data mining, and some data cleaning, analysis, interpretation, and revision of the first draft. TZ: revising the manuscript after submission, reprocessed the data, and proofed the manuscript. BL: study design, funding acquisition, revision of the first draft, review, and submission. All authors had full access to the data in the study, took responsibility for the integrity of the data, and the accuracy of the data analysis. To ensure that any concerns about the accuracy or integrity of any portion of the work are duly examined and addressed, all the authors agree to accept responsibility for all aspects of the work.

## References

[1] de Martino M Pantuck AJ Hofbauer S Waldert M Shariat SF Belldegrun AS. Prognostic impact of preoperative neutrophil-to-lymphocyte ratio in localized nonclear cell renal cell carcinoma. J Urol. (2013) 190:1999–2004. 10.1016/j.juro.2013.06.082 23831313

[2] Huang J Dahl DM Dong L Liu Q Cornejo K Wang Q. Preoperative neutrophil-to-lymphocyte ratio and neutrophilia are independent predictors of recurrence in patients with localized papillary renal cell carcinoma. Biomed Res Int. (2015) 2015:891045. 10.1155/2015/891045 26448948 PMC4573887

[3] Capone M Giannarelli D Mallardo D Madonna G Festino L Grimaldi AM. Baseline neutrophil-to-lymphocyte ratio (NLR) and derived NLR could predict overall survival in patients with advanced melanoma treated with nivolumab. J Immunother Cancer. (2018) 6:74. 10.1186/s40425-018-0383-1 30012216 PMC6048712

[4] Zahorec R. Ratio of neutrophil to lymphocyte counts–rapid and simple parameter of systemic inflammation and stress in critically ill. Bratisl Lek Listy. (2001) 102:5–14. 11723675

[5] Ponti G Maccaferri M Ruini C Tomasi A Ozben T. Biomarkers associated with COVID-19 disease progression. Crit Rev Clin Lab Sci. (2020) 57:389–99. 10.1080/10408363.2020.1770685 32503382 PMC7284147

[6] Wijeratne T Sales C Karimi L Crewther SG. Acute ischemic stroke in COVID-19: a case-based systematic review. Front Neurol. (2020) 11:1031. 10.3389/fneur.2020.01031 33101164 PMC7546832

[7] Zahorec R Hulin I Zahorec P. Rationale use of neutrophil-to-lymphocyte ratio for early diagnosis and stratification of COVID-19. Bratisl Lek Listy. (2020) 121:466–70. 10.4149/BLL_2020_077 32989997

[8] Guthrie GJ Charles KA Roxburgh CS Horgan PG McMillan DC Clarke SJ. The systemic inflammation-based neutrophil-lymphocyte ratio: experience in patients with cancer. Crit Rev Oncol Hematol. (2013) 88:218–30. 10.1016/j.critrevonc.2013.03.010 23602134

[9] Templeton AJ McNamara MG Šeruga B Vera-Badillo FE Aneja P Ocaña A. Prognostic role of neutrophil-to-lymphocyte ratio in solid tumors: a systematic review and meta-analysis. J Natl Cancer Inst. (2014) 106:dju124. 10.1093/jnci/dju124 24875653

[10] Tan TP Arekapudi A Metha J Prasad A Venkatraghavan L. Neutrophil-lymphocyte ratio as predictor of mortality and morbidity in cardiovascular surgery: a systematic review. ANZ J Surg. (2015) 85:414–9. 10.1111/ans.13036 25781147

[11] Vaughan-Shaw PG Rees JR King AT. Neutrophil lymphocyte ratio in outcome prediction after emergency abdominal surgery in the elderly. Int J Surg. (2012) 10:157–62. 10.1016/j.ijsu.2012.02.010 22361307

[12] Cook EJ Walsh SR Farooq N Alberts JC Justin TA Keeling NJ. Post-operative neutrophil-lymphocyte ratio predicts complications following colorectal surgery. Int J Surg. (2007) 5:27–30. 10.1016/j.ijsu.2006.05.013 17386911

[13] Forget P Moreau N Engel H Cornu O Boland B De Kock M. The neutrophil-to-lymphocyte ratio (NLR) after surgery for hip fracture (HF). Arch Gerontol Geriatr. (2015) 60:366–71. 10.1016/j.archger.2014.11.008 25488015

[14] Da Silva M Cleghorn MC Elnahas A Jackson TD Okrainec A Quereshy FA. Postoperative day one neutrophil-to-lymphocyte ratio as a predictor of 30-day outcomes in bariatric surgery patients. Surg Endosc. (2017) 31:2645–50. 10.1007/s00464-016-5278-y 27743125

[15] Faria SS Fernandes Jr PC Silva MJ Lima VC Fontes W Freitas-Junior R. The neutrophil-to-lymphocyte ratio: a narrative review. Ecancermedicalscience. (2016) 10:702. 10.3332/ecancer.2016.702 28105073 PMC5221645

[16] Horne BD Anderson JL John JM Weaver A Bair TL Jensen KR. Which white blood cell subtypes predict increased cardiovascular risk? J Am Coll Cardiol. (2005) 45:1638–43. 10.1016/j.jacc.2005.02.054 15893180

[17] Balta S Celik T Mikhailidis DP Ozturk C Demirkol S Aparci M. The relation between atherosclerosis and the neutrophil-lymphocyte ratio. Clin Appl Thromb Hemost. (2016) 22:405–11. 10.1177/1076029615569568 25667237

[18] Shah AD Denaxas S Nicholas O Hingorani AD Hemingway H. Neutrophil counts and initial presentation of 12 cardiovascular diseases: a CALIBER cohort study. J Am Coll Cardiol. (2017) 69:1160–9. 10.1016/j.jacc.2016.12.022 28254179 PMC5332591

[19] von Elm E Altman DG Egger M Pocock SJ Gøtzsche PC Vandenbroucke JP. The strengthening the reporting of observational studies in epidemiology (STROBE) statement: guidelines for reporting observational studies. Lancet. (2007) 370:1453–7. 10.1016/S0140-6736(07)61602-X 18064739

[20] Riley RD Ensor J Snell KIE Jr FEH Martin GP Reitsma JB. Calculating the sample size required for developing a clinical prediction model. BMJ. (2020) 368:m441. 10.1136/bmj.m441 32188600

[21] Gutierrez CS Passos SC Castro SMJ Okabayashi LSM Berto ML Lorenzen MB. Few and feasible preoperative variables can identify high-risk surgical patients: derivation and validation of the Ex-Care risk model. Br J Anaesth. (2021) 126:525–32. 10.1016/j.bja.2020.09.036 33127046

[22] Muggeo VM. Estimating regression models with unknown break-points. Stat Med. (2003) 22:3055–71. 10.1002/sim.1545 12973787

[23] Steyerberg EW Vickers AJ Cook NR Gerds T Gonen M Obuchowski N. Assessing the performance of prediction models: a framework for traditional and novel measures. Epidemiology. (2010) 21:128–38. 10.1097/EDE.0b013e3181c30fb2 20010215 PMC3575184

[24] Silberman S Abu-Yunis U Tauber R Shavit L Grenader T Fink D. Neutrophil-lymphocyte ratio: prognostic impact in heart surgery. Early outcomes and late survival. Ann Thorac Surg. (2018) 105:581–6. 10.1016/j.athoracsur.2017.07.033 29132702

[25] Jackson SM Perry LA Borg C Ramson DM Campbell R Liu Z. Prognostic significance of preoperative neutrophil-lymphocyte ratio in vascular surgery: systematic review and meta-analysis. Vasc Endovasc Surg. (2020) 54:697–706. 10.1177/1538574420951315 32840176

[26] Perry LA Liu Z Loth J Penny-Dimri JC Plummer M Segal R. Perioperative neutrophil-lymphocyte ratio predicts mortality after cardiac surgery: systematic review and meta-analysis. J Cardiothorac Vasc Anesth. (2022) 36:1296–303. 10.1053/j.jvca.2021.07.001 34404595

[27] Becher RD Hoth JJ Miller PR Meredith JW Chang MC. Systemic inflammation worsens outcomes in emergency surgical patients. J Trauma Acute Care Surg. (2012) 72:1140–9. 10.1097/TA.0b013e3182516a97 22673238

[28] Palin RP Devine AT Hicks G Burke D. Association of pretreatment neutrophil-lymphocyte ratio and outcome in emergency colorectal cancer care. Ann R Coll Surg Engl. (2018) 100:308–15. 10.1308/rcsann.2017.0232 29364006 PMC5958849

[29] Gibson PH Croal BL Cuthbertson BH Small GR Ifezulike AI Gibson G. Preoperative neutrophil-lymphocyte ratio and outcome from coronary artery bypass grafting. Am Heart J. (2007) 154:995–1002. 10.1016/j.ahj.2007.06.043 17967611

[30] Coelho NH Coelho A Augusto R Semiao C Peixoto J Fernandes L. Pre-operative neutrophil to lymphocyte ratio is associated with 30 day death or amputation after revascularisation for acute limb ischaemia. Eur J Vasc Endovasc Surg. (2021) 62:74–80. 10.1016/j.ejvs.2021.03.011 34112572

