# Supplementary material for: Assessment of prognostic value of preoperative neutrophil-to-lymphocyte ratio for postoperative mortality and morbidity
Source: Front Med (Lausanne). 2023 Mar 8;10:1102733. doi: 10.3389/fmed.2023.1102733 (PMC10030720; doi:10.3389/fmed.2023.1102733)
Supplement: Supplementary file 1 [file Table_1.docx]

**Supplemental Table 1. Performance statistics between the extended models and old ones**

Model performance was assessed by a combination of the AUROC (higher c-statistic = better discrimination), Brier score (lower values = higher predictive accuracy), Hosmere-Lemeshow goodness-of-fit test and the AIC (lower score = better model fit). NRI represents the change of reclassification ability between the extended model corresponding to each threshold and the model corresponding to threshold 3.6 of NLR.

Abbreviations: ASA: American Society of Anesthesiologists Physical Status, CCI: Charlson Comorbidity Index, SORT: Surgical Outcome Risk Tool, NRI: Net Reclassification Improvement, AUROC: the area under the receiver operating characteristic curve, AIC: Akaike Information Criterion
