# Supplementary figures and images for: Assessment of prognostic value of preoperative neutrophil-to-lymphocyte ratio for postoperative mortality and morbidity
Source: Front Med (Lausanne). 2023 Mar 8;10:1102733. doi: 10.3389/fmed.2023.1102733 (PMC10030720; doi:10.3389/fmed.2023.1102733)

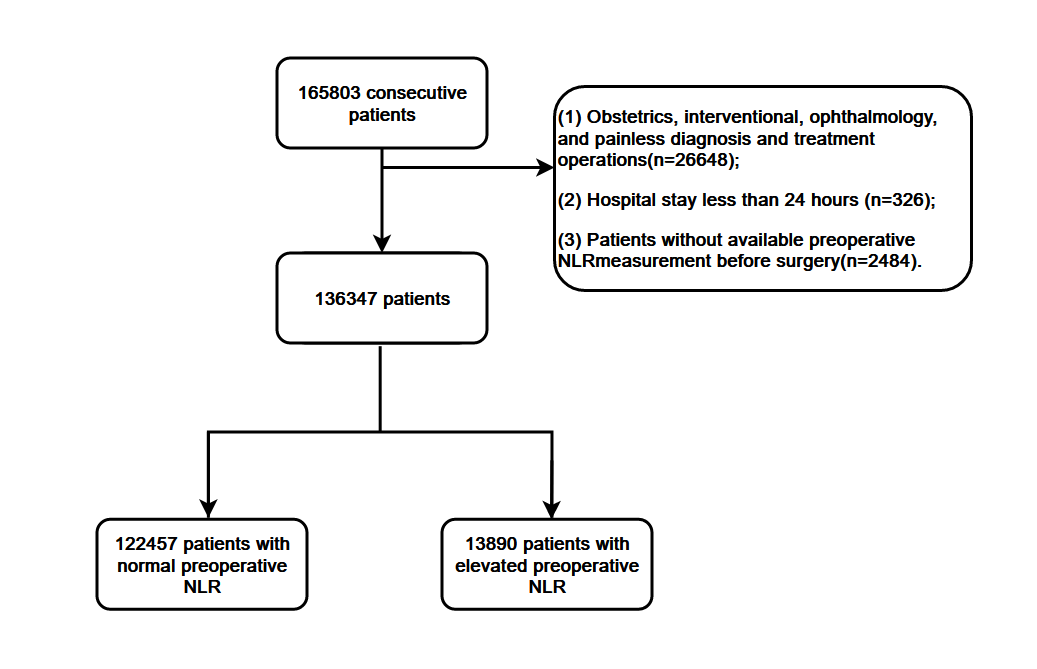

Supplement: Supplementary file 2 [file Image_1.png]

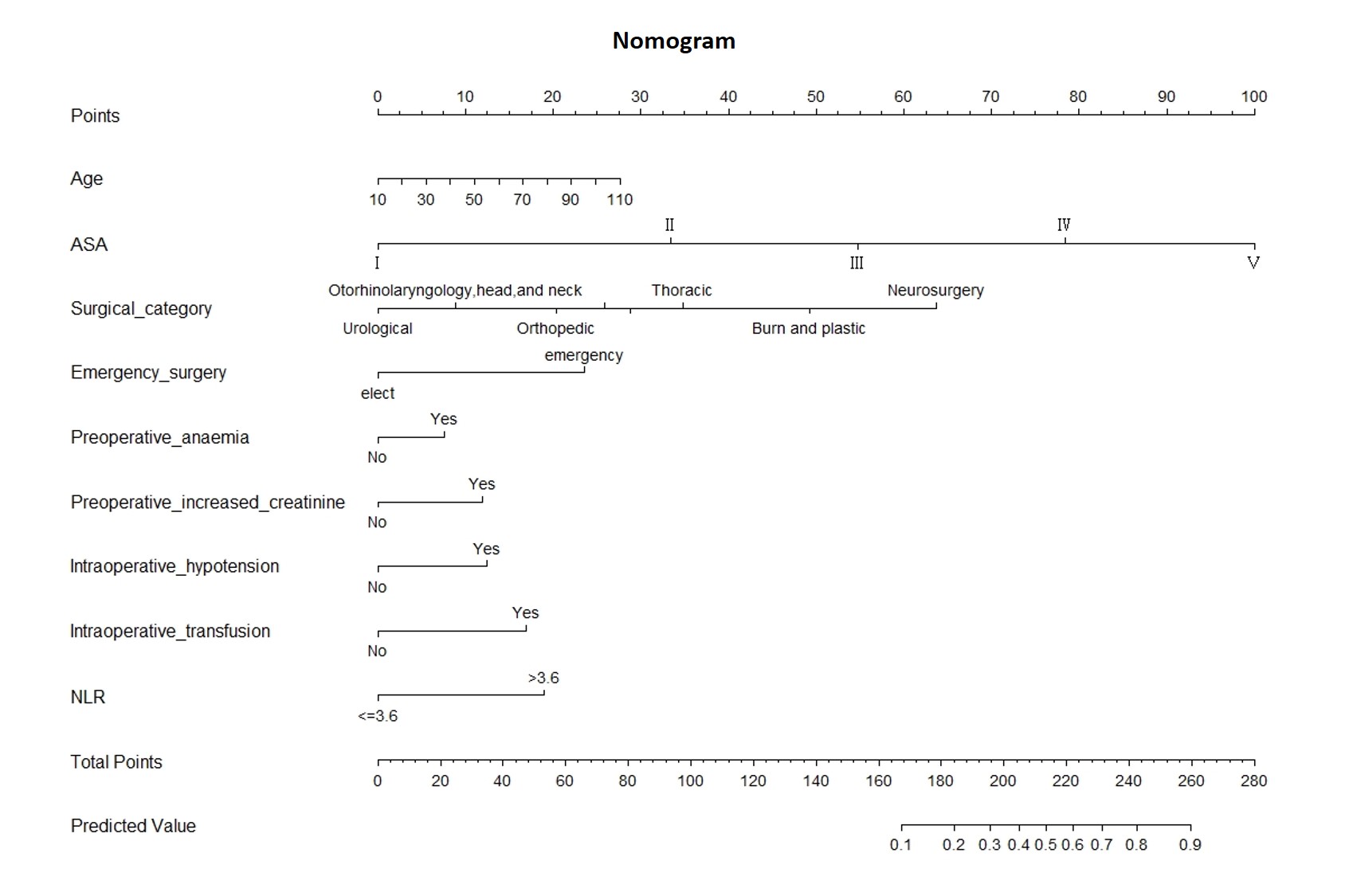

Supplement: Supplementary file 3 [file Image_2.jpg]
